# The Impact of Various Culture Conditions on Human Mesenchymal Stromal Cells Metabolism

**DOI:** 10.1155/2021/6659244

**Published:** 2021-03-01

**Authors:** Pavla Tonarova, Katerina Lochovska, Robert Pytlik, Marie Hubalek Kalbacova

**Affiliations:** ^1^Institute of Pathological Physiology, 1st Faculty of Medicine, Charles University, Prague, Czech Republic; ^2^Biomedical Centre, Faculty of Medicine in Pilsen, Charles University, Pilsen, Czech Republic; ^3^1st Department of Medicine-Department of Haematology, 1st Faculty of Medicine, Charles University and General University Hospital in Prague, Prague, Czech Republic; ^4^Institute of Haematology and Blood Transfusion, 1st Faculty of Medicine, Charles University, Prague, Czech Republic

## Abstract

*In vitro* and *in vivo* analyses are closely connected, and the reciprocal relationship between the two comprises a key assumption with concern to the conducting of meaningful research. The primary purpose of *in vitro* analysis is to provide a solid background for *in vivo* and clinical study purposes. The fields of cell therapy, tissue engineering, and regenerative medicine depend upon the high quality and appropriate degree of the expansion of mesenchymal stromal cells (MSCs) under low-risk and well-defined conditions. Hence, it is necessary to determine suitable alternatives to fetal bovine serum (FBS—the laboratory gold standard) that comply with all the relevant clinical requirements and that provide the appropriate quantity of high-quality cells while preserving the required properties. Human serum (autologous and allogeneic) and blood platelet lysates and releasates are currently considered to offer promising and relatively well-accessible MSC cultivation alternatives. Our study compared the effect of heat-inactivated FBS on MSC metabolism as compared to its native form (both are used as the standard in laboratory practice) and to potential alternatives with concern to clinical application—human serum (allogeneic and autologous) or platelet releasate (PR-SRGF). The influence of the origin of the serum (fetal versus adult) was also determined. The results revealed the key impact of the heat inactivation of FBS on MSCs and the effectiveness of human sera and platelet releasates with respect to MSC behaviour (metabolic activity, proliferation, morphology, and cytokine production).

## 1. Introduction

Fetal bovine serum (FBS) is used widely as a supplement for the culturing of cells and is considered the universal standard component with concern to the majority of culture media. FBS contains key factors for the *in vitro* preservation and successful expansion of cells, e.g., growth factors, vitamins, lipids, hormones, and various proteins that directly or indirectly participate in cell adhesion and growth. Due to its blood origin, FBS is considered the tool that best contributes to the imitation of the natural environment of organisms, and it is critical for both the short-term [1] and long-term [2] maintenance and expansion of cells [3] under in vitro conditions. Nevertheless, FBS as a typical “representative” serum has a number of disadvantages. Firstly, no batch is the same in terms of composition and quality [4], and the fact that it is chemically undefined is detrimental with regard to experimental reproducibility. In addition, a number of analytical methods are negatively affected to various extents by both its components and the differing concentrations thereof. Secondly, FBS is a xenogeneic agent in the human medicine context. Furthermore, FBS can be connected with a high risk of contamination by, e.g., endotoxins, viruses, and prions, despite it is often being heat treated, i.e., heat inactivated prior to its practical application. A single study describes patients who developed antibodies to a single component of fetal calf serum (FCS), which resulted in an arthus-like reaction following the administration of lymphocytes cultivated in a medium supplemented with FCS [5]. Thus, it is not particularly safe for clinical applications nor suitable for the *in vitro* experiments that usually precede them. Thirdly, the FBS collection method is highly controversial and strongly nonethical [6]. Therefore, in view of the significant negative factors, considerable efforts are being made to replace FBS with other alternatives.

The most promising FBS alternatives for use in human medicine comprise human serum (HS), blood platelet lysates (PL) [7], and platelet releasates (PR-SRGF) [8] used either alone or in combination. In contrast to PL, which usually contains all the factors and components of platelets, the PR-SRGF (a platelet releasate-supernatant rich in growth factors) contains only those factors released from activated platelets [6, 9, 10]. The *α*-granules of platelets, particularly, are rich in various adhesive proteins, cytokines, chemokines, and growth factors and are released from the platelets following the activation thereof [11]. Despite their batch-to-batch variability, these alternatives resolve most of the basic problems associated with the application of FBS: they are native to the human organism, easily accessible, and their collection is ethical. In addition, the input materials for the production of these alternatives have for the most part been clinically tested. Although HS, PL, and PR-SRGF represent promising supplements due to their native and human origin, further detailed analysis and studies will be required, and guidelines will have to be set [3].

While the potential of mesenchymal stromal cells (MSCs) to fundamentally extend the options available to regenerative medicine is indisputable, especially with respect to such cells in the undifferentiated state [12, 13], defining the approach to the culturing of MSCs remains a critical factor and a necessary prerequisite for both the study and practical application of these cells. The determination of optimal culturing conditions including the effective expansion and simultaneously maintenance of the natural properties of the cells (self-renewal, multipotency) in the culture remains a challenge for the fields of cell biology and regenerative medicine. Thus, we focused on the characterization of human MSCs cultivated in the presence of heat-modified and non-heat-modified FBS (although both versions are widely used, insufficient attention has, to date, been devoted the difference between them) and alternatives such as human serum of differing origin (autologous and allogeneic) and platelet. All of these culture conditions were compared to a medium supplemented with FBS that remains the standard MSC cultivation supplement.

Our results suggested the significant impact of FBS and FBS alternatives treated in various ways on cell behaviour in terms of growth, metabolism, differentiation routes, colony formation, and cytokine production.

## 2. Materials and Methods

### 2.1. MSCs and Culturing Conditions

Human MSCs were obtained from the bone marrow of healthy donors following signed informed consent and with the approval of the respective ethical committee. Bone marrow mononuclear cells (BMMNCs) were isolated by means of centrifugation with Ficoll-Histopaque medium (Sigma-Aldrich) and initially expanded in alpha-MEM with 10% nonheat inactivated FBS (non-iFBS). MSCs from the initial expansion were passaged once and then frozen. According to the type of experiment (see below), the adherent cells were further cultivated on 75 or 25 cm^2^ flasks, 6-, 12-, 24-, or 96-well plates (TPP, Switzerland) in a standard *α*-MEM medium (Life Technologies, USA) with penicillin (20 U/ml; Sigma-Aldrich, USA) and streptomycin (20 mg/ml; Sigma-Aldrich, USA) and with the selected serum type or alternatives thereof (see Table 1). The heat inactivation of the FBS was performed at 56°C for 30 min (non-iFBS from the same batch was always used as the control).

**Figure 1:**
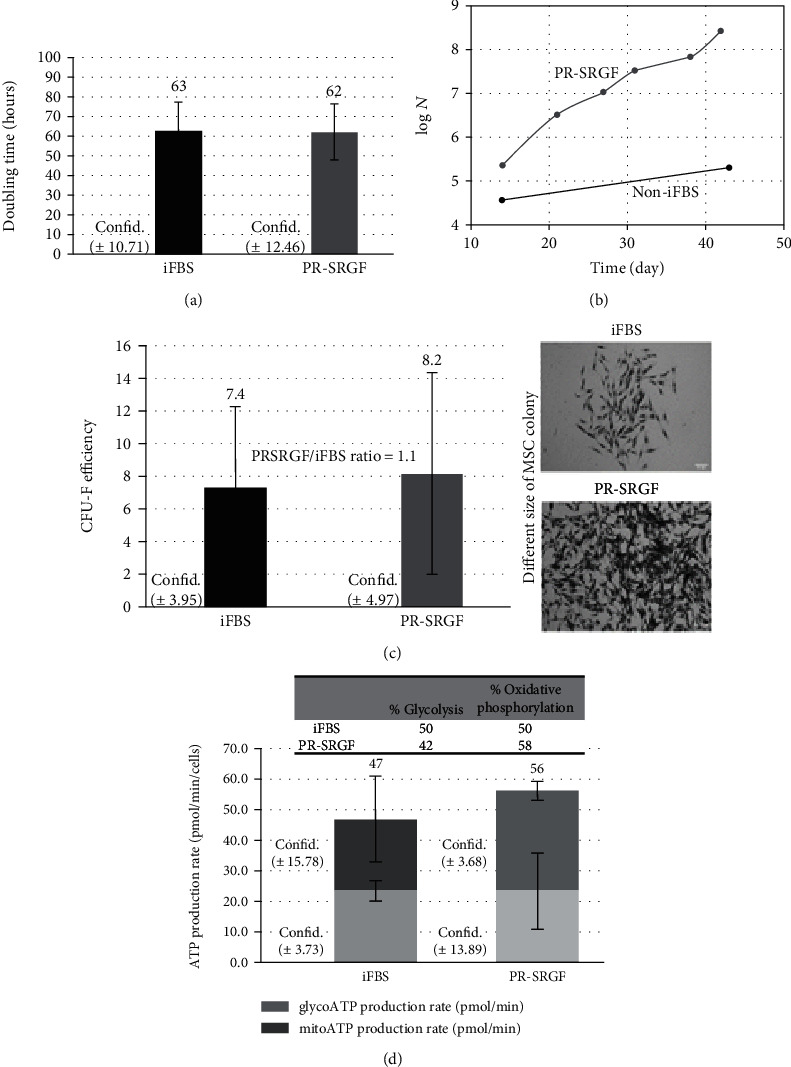
Influence of different media supplements on MSC behaviour. Analyses of MSCs cultivated in media with heat-inactivated FBS (iFBS) and platelet releasate (PR-SRGF): (a) doubling time of MSC during first 48 h of cultivation; (b) long-term proliferation of MSCs during 42 days of cultivation; (c) CFU-F and colony morphology during first 48 h of cultivation (white bars correspond to 232 *μ*m); (d) ATP production rate (pmol/min/cells) and % of glycolysis and oxidative phosphorylation after 24 h of cultivation ((a–d) + (f) in 10% iFBS and 7.5% PR-SRGF; (e) in 10% non-iFBS and 7.5% PR-SRGF). Statistical analysis was done by one factor ANOVA (*p* < 0.05) with post hoc Fischer LSD test (95% confidence interval was determined to verify the similarity/comparability of samples with a nonsignificant difference).

In general, the cells were cultivated at 37°C and in a 5% CO_2_ atmosphere. The experiments were performed using MSCs from healthy donors (*n* = 2‐4) with a passage number of from two to four (the cells were seeded at various concentrations—7 000, 8 000, and 10 000 cells/cm^2^). Bone marrow aspirates were also obtained from patients with various haematological diagnoses (*n* = 5) for the long-term proliferation assay of the MSCs (Figure 1(b)) following signed informed consent. The bone marrow-derived mononuclear cells (BMMNCs) were isolated via centrifugation with Ficoll-Histopaque medium (Sigma-Aldrich) and seeded at a concentration of 1 × 10^5^ cells/cm^2^ in *α*-MEM (Sigma-Aldrich) containing L-glutamine (Gibco Invitrogen) and penicillin/streptomycin (Sigma-Aldrich) supplemented with non-iFBS or 7.5% PR-SRGF (*n* = 4). The cells were incubated at 37°C and in a 5% CO2 atmosphere. The nonadherent cells were washed out 24 hours following seeding. The initial expansion of the adherent cells lasted 14 days, and subsequent passages (maximum of 6 passages) were always performed at 80-90% confluence with TrypLETM (Gibco Invitrogen). The reseeding concentration was 2 600 cells/cm^2^ from the first passaging.

### 2.2. Human Serum Preparation

The human sera, allogenic human serum (allo-HS; a mixture of human plasma sera from unknown donors of various blood groups), autologous human serum (auto-HS; serum from the donor of the MSCs used), and serum derived from an AB blood group donor (AB-HS), were obtained from healthy donors following signed informed consent. The sera were prepared following the clotting of the blood and subsequent centrifugation (3 000 rpm/10 minutes). The human sera were not heat inactivated.

### 2.3. Preparation of PR-SRGF

Two batches of PR-SRGF were prepared from various donors. The chemical activation of the platelets was performed via the addition of CaCl_2_ up to a final concentration of 0.04 M. After one hour of incubation at 40°C followed by centrifugation at 2 200 × g for 10 min, the PR-SRGF was pooled and filtered using a 0.22 *μ*m pore size filter. The aliquots were stored at -20°C pending usage.

### 2.4. CFU-F Analysis

With concern to the colony-forming unit-fibroblast (CFU-F) assay, 1.5 cells/cm^2^ were plated onto a 60 cm^2^ polystyrene Petri dish (TPP, Switzerland) and cultivated for 2 weeks with the exchange of the medium every 3 days. The colonies thus formed were stained by means of crystal violet (Sigma-Aldrich, 0.5% solution in methanol) and counted by the naked eye. Those cell clusters larger than 1 mm in diameter were considered to be CFU-Fs. The CFU-F efficiency in the graphs represents the percentage of colonies that were formed from the initial number of seeded cells.

### 2.5. Determination of the Metabolic Activity

Cells were seeded on 96-well plates (TPP, Switzerland) at concentrations of 8 000 cells/cm^2^ and 10 000 cells/cm^2^ in the culture medium (see MSCs and Culture Conditions) supplemented with the selected serum type or alternatives thereof (10% iFBS and non-iFBS and 7.5% PR-SRGF). A metabolic activity test (Cell Titer 96 AQueous One Solution Cell Proliferation Assay, MTS, Promega, USA) was performed according to the standard protocol of the manufacturer (the reduction of the MTS reagent to a coloured formazan product was induced by metabolically active cells) after 4, 24, and 48 h. The absorbance was measured directly in a 96-well plate using a multidetection microplate reader (LabSystem MultiSkan MS, Finland). The results were expressed relative to the control cells and normalised to the number of counted cells or the amount of DNA content based on the fluorescence intensity in the samples using CyQUANT NF Cell proliferation Assay Kit (Invitrogen).

### 2.6. Determination of the Doubling Time

MSCs were seeded on 6-well plates (TPP, Switzerland) at a concentration of 10 000 cells/cm^2^ in the standard culture medium supplemented with the selected serum type or alternatives thereof. After 4 h, 24 h, and 48 h of culturing, the cells were harvested (300 *μ*l of trypsin was used) and counted in a Bürker chamber. The doubling time of the cells was determined using the Cell Calculator available online (http://www.doubling-time.com/compute.php?lang=en).

### 2.7. Osteogenic Differentiation of the MSCs in iFBS and Non-iFBS

MSCs were seeded at a concentration of 8 000 cells/cm^2^ in 6-well plate (TPP, Switzerland) in the standard expansion *α*-MEM medium (Life Technologies, USA) with 10% heat-inactivated FBS (PAA, Austria), penicillin (20 U/ml; Sigma-Aldrich, USA), and streptomycin (20 mg/ml; Sigma-Aldrich, USA). After attaining 90% confluency, the cells were treated with osteo-differentiation medium for 21 days (standard *α*-MEM medium (Life Technologies, USA)) with 10% heat-inactivated or noninactivated FBS (PAA, Austria), penicillin (20 U/ml; Sigma-Aldrich, USA), and streptomycin (20 mg/ml; Sigma-Aldrich, USA) supplemented with 0.5 mM sodium L-ascorbate, 10 mM glycerol-phosphate, and 0.1 *μ*M dexamethasone that was replaced every 3 days with freshly supplemented medium. The cells were then stained using the von Kossa staining method and visualised by means of light microscopy.

### 2.8. Determination of the Metabolism

The energy metabolism of the cells (glycolysis and oxidative phosphorylation) was analysed using the Agilent Seahorse XFp Extracellular Flux analyser (Agilent, USA) and XF Real-Time ATP Rate Assay (Seahorse XFp Real-Time ATP Rate Assay Kit, Agilent, USA). The MSCs were cultivated in the standard medium supplemented with 10% iFBS or 7.5% PR-SRGF for 8 days in 25 cm^2^ flasks (TPP, Switzerland; seeded at 5 200 cells/cm^2^). The cells were then harvested and reseeded (7.000 c/well) on 8-well XFp cell culture miniplates (Agilent, USA) in the appropriate medium for 24 h. 1 h prior to the analysis, the seeded cells were treated with fresh Seahorse XF DMEM medium, pH 7.4, supplemented with 10 mM Seahorse XF Glucose, 1 mM Seahorse XF Pyruvate, and 2 mM Seahorse XF L-Glutamine and incubated at 37°C for 1 h. The analysis was performed using hydrated XFp Cartridges supplemented with injection solutions (Oligomycin and Rotenone/Antimycin A). Following the analysis in the Seahorse analyser (total ATP production rate, glycolytic or mitochondrial ATP production rate, % of glycolysis or oxidative phosphorylation), the cells were fixed with 4% paraformaldehyde in PBS at 25°C for 15 min and stained by means of DAPI at 37°C for 15 min (1 : 1 000; Sigma-Aldrich, USA). Images of the cell nuclei were taken using an Eclipse Ti-S microscope with a DS-Qi1Mc digital camera (Nikon, Japan) and a 4x lens. The cell nuclei were counted using the Cell Profiler software (USA). The Seahorse data was then normalised to the resulting cell numbers. The experiments were repeated three times in total (all the experiments were performed in triplicates).

### 2.9. FACS Analysis

The following surface markers were selected for the FACS analysis of the MSCs: CD14-FITC (Dako; dil. 1 : 50), CD45-FITC (Dako; dil. 1 : 50), CD73-PE (Biolegend, clone AD2; dil. 1 : 50), and CD-90-FITC (Dako; dil. 1 : 50). After 72 h of culturing, the MSCs were trypsinized and resuspended in the standard medium, whereupon 30 000 cells/sample were analysed. The cells were incubated with the appropriate antibody for 30 min at 4°C, then washed and resuspended in 300 *μ*l of PBS. The FACS analysis was performed on a BD FACS CANTO device.

### 2.10. Light Microscopy

Phase contrast images of the cells were obtained using an Eclipse Ti-S microscope with a DS-Qi1Mc digital camera (Nikon, Japan). The images were acquired with a 10x lens and adjusted using the ImageJ software (Rasband, W.S., ImageJ, US National Institutes of Health, Bethesda, Maryland, USA).

### 2.11. Fluorescence Staining of the Cells and Confocal Microscopy

The cells were seeded on a 4-well chamber slide (Thermo Scientific™ Nunc™ Lab-Tek™ II Chamber Slide and trade, USA) and cultivated for 48 h. With respect to the mitochondrial staining, the cells were incubated with MitoTracker Red CMX Ros (Invitrogen, USA) at 37°C and in a 5% CO_2_ atmosphere for 30 min. They were then fixed via 4% paraformaldehyde in PBS at RT for 15 min, permeabilised using 0.1% Triton X-100 in PBS (Sigma-Aldrich, USA) at RT for 20 min, and stained using DAPI at 37°C for 15 min (1 : 1 000; Sigma-Aldrich, USA). Confocal images of the cells were acquired using a Leica SP8X microscope (Leica Microsystems, Germany) equipped with a confocal scanning head, a Leica DFC365 FX monochrome digital CCD camera, an HC PL APO CS2 63x/1.40 OIL objective, a 405 nm excitation laser, and a white light laser (WLL; EX 579/EM 599; hybrid detector) (Leica Microsystems, Germany). The images were rendered using the LasX software (Leica Microsystems, Germany).

### 2.12. Cytokine Analysis

The cytokine content in the cell media was analysed using Human Cytokine Antibody Array for 42 targets (Abcam, UK). 7 000 cells/cm^2^ were preseeded in the standard medium (alpha MEM supplemented with 10% iFBS) for 12 hours at 37°C and in a 5% CO_2_ atmosphere, whereupon fresh alpha MEM supplemented with 10% iFBS or 7.5% PR-SRGF was added to the cells for an additional 72 h. Only those media (without cell culturing) incubated under the same conditions as the cells were used as the controls. Subsequently, the culture media were collected and stored at -20°C. The total protein concentration in the conditioned medium was determined using the Pierce™ BCA Protein Assay Kit (Thermo Fisher Scientific, USA); the media were diluted to a final volume of 1 ml. Finally, 1 ml of each culture medium was applied per one cytokine membrane. The analysis was repeated twice and was performed according to the manufacturer's recommended protocol. WB digital imaging (ChemiDoc MP, BioRad) and the Image Lab software (BioRad, Czech Republic) were used for the determination of the final cytokine content.

### 2.13. Capillary ELFO Analysis

The capillary ELFO analysis of the nondiluted supplements, iFBS (two different batches), PR-SRGF (two different batches), human AB serum provided by Sigma-Aldrich (AB-HS (Sigma)), and AB-HS (home-prepared), was performed using the CAPILARYS 2 Flex-Piercing, type 1 227 device (Sebia, France) according to the standards and standard protocols applied in the accredited Laboratory of Clinical Biochemistry. The results of the analysis were compared to the serum reference range (the routinely applied human serum standards: albumin, alpha-1-globulins (alpha 1), alpha-2-globulins (alpha 2), beta-1-globulins (beta 1), beta-2-globulins (beta 2), and gamma-globulins (gamma)).

### 2.14. Statistical Analysis

The statistical results were obtained from two to four independent experiments performed at least in duplicates. The data was statistically analysed by means of one factor ANOVA with or without the post hoc Fischer LSD test. The values obtained were tested for statistically significant differences at an alpha level of 0.05. The statistical evaluation was performed using the Statistica software (StatSoft CR, s.r.o.).

## 3. Results

### 3.1. The Impact of Heat-Inactivated versus Noninactivated FBS on Cell Metabolism and Proliferation

**Figure 2:**
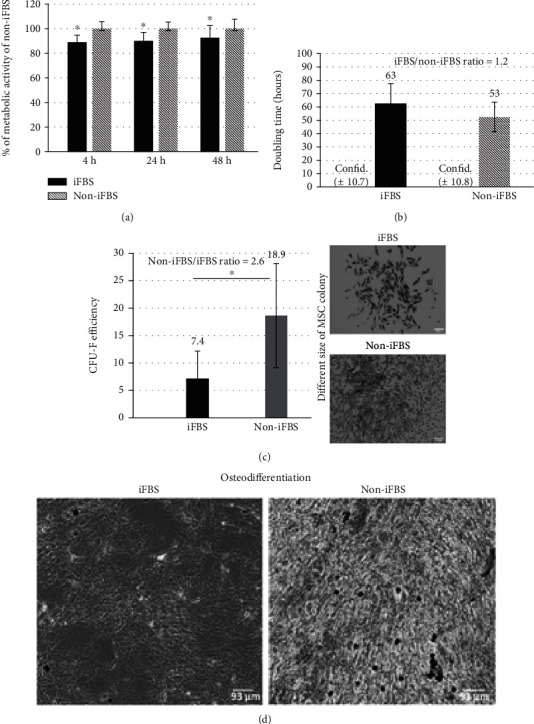
Influence of differently treated FBS on MSC behaviour. Analyses of hMSCs cultivated in media with heat-inactivated FBS (iFBS) and heat noninactivated FBS (non-iFBS) after 48 h of cultivation (a, b) or 14 days (c) or 21 days (d): (a) metabolic activity; (b) doubling time; (c) CFU-F and colony morphology (white bars correspond to 232 *μ*m); (d) osteo-differentiation of hMSCs cultivated in osteo-differentiation media supplemented with iFBS and non-iFBS (used von Kossa staining) ((a) Analysed in 5% iFBS and 5% non-iFBS; (b–d) analysed in 10% iFBS and 10% non-iFBS). Statistical analysis was done by one factor ANOVA (*p* < 0.05) with post hoc Fischer LSD test. ^∗^Significant difference (*p* < 0.05) between iFBS and non-iFBS (95% confidence interval was determined to verify the similarity/comparability of samples with a nonsignificant difference).

The impact of heat-inactivated (iFBS) and noninactivated FBS (non-iFBS) on the metabolic activity and proliferation of MSCs was studied. Those MSCs cultivated up to 48 h either with 10% of iFBS or with 10% of non-iFBS exhibited significant differences in their metabolic activities (Figure 2(a)). In the presence of non-iFBS, the MSCs evinced a higher metabolic activity than in the iFBS-supplemented medium. The difference in their doubling time (Figure 2(b)) was not significant due to differences between the patients; however, an increase in MSC proliferation was indicated in the iFBS-supplemented medium, and the ratio of the doubling time of the cells cultivated in iFBS to the non-iFBS-supplemented medium was 1.2.

The ability of the MSCs to form colonies (CFU-F) was found to be significantly reduced in the iFBS medium compared to the non-iFBS medium (Figure 2(c)). The significant difference was confirmed via the determination of a CFU-F efficiency ratio of 2.6. Moreover, the MSC colonies cultivated over 14 days in the non-iFBS were seen to be more cell dense and larger than the colonies raised in the iFBS-supplemented medium.

Furthermore, the markedly reduced ability of the MSCs to osteo-differentiate in the presence of the iFBS was apparent compared to the cells in the presence of the non-iFBS (Figure 2(d)).

### 3.2. Impact of the Various Types of Sera on the Metabolic Activity of the MSCs

**Figure 3:**
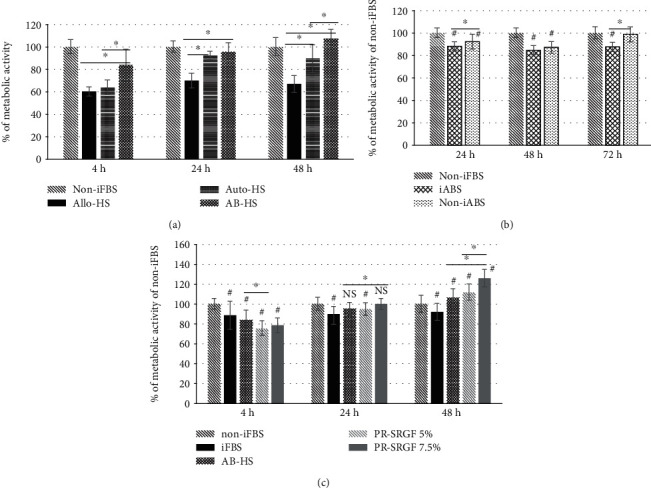
Metabolic activity of MSCs cultivated in differently supplemented media. (a) Cells in the media supplemented with 5% allogenic human serum (allo-HS), 5% autologous human serum (auto-HS), and 5% human serum derived from donor of the AB blood group (AB-HS) (results compared to % of non-iFBS). All the results were significantly different to non-iFBS (*p* < 0.05). (b) Metabolic activity of MSCs cultivated in media with non-iFBS, heat inactivated adult bovine serum (iABS), or noninactivated adult bovine serum (non-iABS). #Significant difference between non-iFBS and samples (*p* < 0.05). (c) Cells in the media supplemented with 5% heat inactivated FBS (iFBS), 5% human AB serum (AB-HS), and 5% or 7.5% platelet releasate (PR-SRGF) supplement after 4–48 h (results compared to % of non-iFBS). NS: nonsignificant difference to non-iFBS control. ^∗^Significant difference between samples. #Significant difference between non-iFBS and samples (*p* < 0.05), based on one factor ANOVA with post hoc Fischer LSD test.

The next stage focused on the impact of the various types of sera on the metabolic activity of the MSCs. The media supplemented with adult noninactivated (i) allogeneic human serum (allo-HS; a mix of human plasma sera from unknown donors with differing blood groups), (ii) autologous human serum (auto-HS, serum from the same donor as the origin of the MSCs), and (iii) serum derived from the donor with the AB blood group (AB-HS) were tested against the medium supplemented with the non-iFBS (control—its metabolic activity was considered to be 100%) (Figure 3(a)). In contrast to the non-iFBS, the metabolic activity of all the tested cells cultivated in all the FBS adult alternatives was found to be significantly lower, with the exception of the 5% AB-HS, which was comparable to the non-iFBS control as soon as after 24 h and significantly higher after 48 h. The significant inhibitory effect of allo-HS on the MSCs in contrast to that of AB-HS and auto-HS was apparent for the whole of the cultivation period, with the metabolic activity of the cells cultivated in the allo-HS attaining only 60-70% of the control. A reduction in the metabolic activity to below 75% of the control is generally considered to be an “inhibitory or cytotoxic” level [14]. On the other hand, while the metabolic activity of the MSCs cultivated in the medium with auto-HS was initially also found to have decreased, it eventually approached the control level. Moreover, the data revealed that the AB-HS and auto-HS supported the metabolism of the MSCs significantly more than did the allo-HS, and the metabolic activity of the MSCs was seen to be elevated in the AB-HS for the whole of the cell cultivation period as opposed to that of the auto-HS.

Further, with respect to the differing origin of the tested sera, we compared the metabolic activity of the MSCs cultivated in fetal bovine serum compared to that of the adult bovine serum (BS) (Figure 3(b)). The reduction in the cell metabolism in the adult bovine serum was observed to be significant in almost all cases. Moreover, the similar effect of serum heat inactivation (the reduction in the metabolic activity in the iBS compared to that in the non-iBS) was apparent, as shown in Figure 2. It is important to note that since this study used different batches over the course of experimentation, the general phenomenon of the batch-to-batch variation of the FBS and its alternatives should also be borne in mind [15, 16].

### 3.3. Impact of the PR-SRGF Serum Alternative on the MSCs

The next stage involved the testing of the effects of a “platelet releasate-supernatant rich in growth factors” (PR-SRGF, currently considered to be a valuable alternative to FBS) on the behaviour of the MSCs.

A comparison of the metabolic activities of MSCs treated with various non-iFBS alternatives (iFBS, AB-HS, and PR-SRGF at two concentrations, 5% and 7.5%) is provided in Figure 3(c). In the early phase of cultivation (4 h), the metabolic activity of the MSCs in all the tested supplements was observed to be significantly lower than that of the control. However, over time, the metabolic activity of the cells cultivated in the media supplemented with AB-HS and both concentrations of PR-SRGF (the higher concentration was seen to be more supportive) reached the level of the control (at 24 h) and even exceeded it dependent on the concentration (at 48 h).

We then focused on the impact of PR-SRGF on the doubling time (Figure 1(a)) and long-term proliferation (Figure 1(b)) of the cells in comparison to the FBS in the cultivation medium. While the doubling time analysis after 48 h indicated no statistically significant differences between the iFBS and the PR-SRGF (Figure 1(a)), a significant increase in cell proliferation was apparent after 42 days with concern to the cells cultivated in the PR-SRGF-supplemented medium. Significantly higher yields/numbers of MSCs in the medium with PR-SRGF were observed as early as at the commencement of cultivation (day 12), and at the end of the experiment, the number of cells cultured in the PR-SRGF was seen to be 45 000 times higher (resulting in 6 passages of the cells in 42 days) than that of the cells cultured in the non-iFBS (resulting in only 2 passages of the cells in 42 days).

The analysis of the CFU-F of the MSCs cultured under both conditions for 14 days revealed that although the CFU-F of the cells in the PR-SRGF was higher than that of the cells cultured in the iFBS (the PR-SRGF/iFBS ratio was 1.1); the difference was not statistically significant (Figure 1(c)). Interestingly, in contrast to the CFU-F efficiency, the size of the colonies differed significantly (see the images in Figure 1(c)). Compared to the colonies in the iFBS, those in the PR-SRGF medium were larger and much denser.

The study also included the performance of the FACS analysis of the surface markers of the MSCs cultured in the presence of iFBS and PR-SRGF (Supp. 1). Whereas CD73 and CD90 (typical MSC surface markers) were determined on the cells cultivated in these media, there was no indication of CD14 and CD45 [17]. The presence of PR-SRGF did not fundamentally alter the expression of the selected markers compared to the presence of iFBS—the positive markers evinced more than 95% positivity while the negative markers evinced less than 1% positivity.

**Figure 4:**
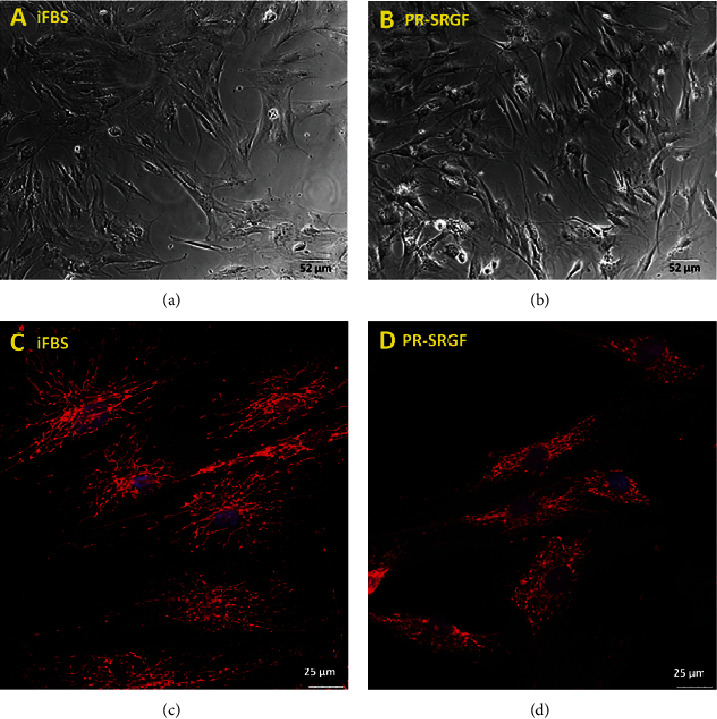
(a, b) Light microscopy of living unstained MSCs (10x lens) cultivated for 48 h in medium supplemented with 10% heat inactivated FBS (iFBS) (a) and 7.5% PR-SFGF (b). (c, d) Confocal images of mitochondrial network of fixed MSC (63x lens) cultivated for 48 h in medium supplemented with 10% heat inactivated FBS (iFBS) (c) and 7.5% PR-SFGF (d).

The analysis was also performed of the morphology of the MSCs cultivated in the media supplemented with PR-SRGF compared to that of the media supplemented with iFBS; differences were apparent even after just 48 h of cultivation (Figure 4). While the cells in the iFBS medium were observed to be flatter and with a spindle-shaped morphology (Figure 4(a)), the MSCs cultivated in the PR-SRGF medium (Figure 4(b)) were significantly more convex in the nuclei region and thinner. Moreover, the confocal microscopy of the mitochondrial network of the MSCs cultured under both conditions revealed additional apparent differences (Figures 4(c) and 4(d)). While the mitochondrial network of the MSCs cultured in the iFBS-supplemented medium (Figure 4(c)) appeared to be spread out with thick, long, single fibres densely located around the cell nucleus in a star-like pattern (perinuclear region > peripheral region), the mitochondrial network of the MSCs cultured in the PR-SRGF-supplemented medium (Figure 4(d)) was more compact with shrunken ball-like structures evenly distributed over the cells (perinuclear region = peripheral region). The differing mitochondrial morphological pattern thus suggested the differing impact of PR-SRGF on the metabolism of the MSCs compared to that of the iFBS; this was supported by the analysis of the metabolic activity of the MSCs (Figure 3(c)), i.e., the cells incubated in the PR-SRGF-supplemented medium evinced an approximately 30% higher metabolic activity than the cells incubated in the iFBS-supplemented medium.

Consequently, the more detailed analysis of the metabolisms of the MSCs cultivated in the PR-SRGF and FBS was performed (Figure 1(d)). The ratio of the glycolysis-formed and oxidative phosphorylation-formed ATP as well as the ATP formation rate in these cells was compared by means of Seahorse equipment.

While the cells incubated in the iFBS for 24 h maintained a similar ratio between oxidative phosphorylation and glycolysis, the cells cultivated in the PR-SRGF produced ATP to an approximately 16% greater extent via oxidative phosphorylation than via glycolysis. In addition, the PR-SRGF increased the total ATP formation rate in the cells by approximately 20% compared to the iFBS (47 pmol ATP/min in iFBS vs. 56 pmol ATP/min in PR-SRGF).

**Figure 5:**
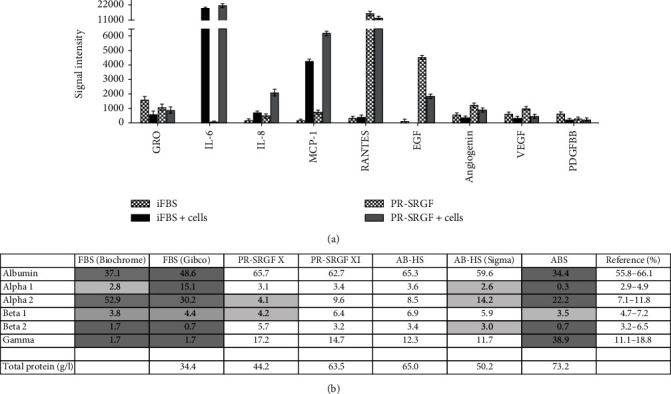
(a) Analysis of selected cytokines in the medium supplemented with 10% iFBS and 7.5% PRSRGF after cultivation with MSC (iFBS + cells, PR − SRGF + cells) or without cell cultivation (iFBS, PR-SRGF) after 72 hours. (b) Determination of protein fractions in nondiluted iFBS, PR-SRGF, AB-HS, AB-HS (Sigma), and ABS (adult bovine serum) (without cell cultivation) by capillary ELFO analysis compared to the human reference standard (albumin, alpha-1-globulins, alpha-2-globulins, beta-1-globulins, beta-2-globulins, gamma-globulins). Light grey: out of the reference range; dark grey: extremely out of the reference range.

Finally, the analysis was performed of the cytokine content in the media supplemented with 10% iFBS and 7.5% PR-SRGF after 72 h of cultivation with the cells (iFBS + cells, PR − SRGF + cells) and without cells as controls (iFBS, PR-SRGF). Of a total number of 42 tested cytokines, the most noticeable differences were detected at the level of the following cytokines (Figure 5(a)): GRO (growth regulated oncogene), IL-6 (interleukin 6), IL-8 (interleukin 8), MCP-1 (monocyte chemoattractant protein-1), RANTES (regulated upon activation, normal T cell expressed and secreted; CCL5), EGF (*epidermal growth factor*), angiogenin, VEGF (*vascular endothelial growth factor*), and PDGF-BB (platelet-derived growth factor-BB). In general, the overall cytokine levels were found to be markedly lower in the iFBS-supplemented medium with and without cells than in the PR-SRGF-supplemented medium, with just two exceptions—the GRO and PDGF-BB. The PR-SRGF-supplemented medium *per se* contained higher levels of RANTES and EGF and slightly more elevated levels of VEGF and angiogenin than did the medium supplemented with iFBS. Conversely, IL-6, IL-8, and MCP-1 were undoubtedly produced by the cells since the levels thereof were elevated only in those samples containing cells. Interestingly, certain cytokines were reduced in the presence of cells, i.e., GRO, angiogenin, and VEGF (in both supplements); EGF and RANTES (in the PR-SRGF); and PDGF-BB (in the iFBS), thus suggesting their degradation via cell consumption.

### 3.4. Protein Content Analysis of the Sera and Supplements

With the aim of investigating the possible impact of differing sera and supplements from the clinical point of view, we performed the analysis of nondiluted samples of FBS, PR-SRGF, and human sera by means of capillary electrophoresis (Figure 5(b)), which is the standard procedure applied for the protein analysis of human serum in clinical practice. All the tested supplements were compared with the reference human serum standard set in the diagnostic laboratory. The results showed that while both batches of PR-SRGF and AB-HS were similar to the human serum reference standard, the two batches of FBS differed from the human reference. Interestingly, the albumin fraction was significantly reduced in the FBS in contrast to that of the human samples. On the other hand, significantly elevated fractions of alpha-2 globulins were determined in both batches of the FBS in contrast to all the human samples. Moreover, we also compared the proteins in the fetal and adult bovine sera (FBS versus ABS) and discovered protein levels that differed from the human standard in both cases; however, an elevated level of gamma globulins was apparent in the ABS (also higher than in the adult human samples) related to the enhanced presence of various antibodies that are not present in fetal serum.

## 4. Discussion

### 4.1. The Impact of Heat-Inactivated versus Noninactivated FBS on Cell Metabolism and Proliferation

The results presented in Figures 2(a)–2(c) indicated the reduced doubling time, cell metabolic activity, and CFU-F efficiency of bone marrow-derived hMSCs in the medium supplemented with heat-inactivated FBS compared to the cells cultivated in the noninactivated FBS. A significant decrease in the cell proliferation of bone marrow-derived MSCs in iFBS after 14 days was also recorded by Nimura et al. [18]; however, in contrast to non-iFBS, the synovial MSCs in the same study were not affected by iFBS. Thus, Nimura's results evinced differences not only in terms of serum modification but also concerning the tissue origin of MSCs under the same conditions. In addition, he raised questions with respect to the enhanced variability of MSCs of the same origin linked to differing MSC donors, which was, in many cases, also apparent from the presented results and the high standard deviations. Conversely, however, Bruinink et al. observed a similar proliferation rate of bone marrow-derived MSCs cultivated in the presence of FBS regardless of the heat modification thereof. However, he did demonstrate the influence of FBS modification related to other cell types (primary bone cells) [19]. In summary, while all these results indicate that the heat inactivation of FBS is capable of markedly affecting the behaviour of cells, its impact may be significantly affected by the origin of the cells and cell donor variability [20, 21] and the cell harvesting method, number of cell passages, cell cultivation time [22], and quality of the serum.

In addition, the markedly reduced ability was observed of MSCs to osteo-differentiate in the presence of heat-inactivated FBS (Figure 2(d)). These results are supported by other studies that have suggested the role of heat labile serum factors (e.g., complement compounds and various mitogenic factors such as growth factors) [19, 23]. This fact alone also explains the differing responses of the various cell types (with respect to the activity of the cell cycle and metabolism) and the variability of the FBS batches. As has been previously demonstrated, the protein profiles of inactivated and noninactivated sera exhibit significant qualitative and quantitative differences [24]. This analysis would appear to suggest that the serum conditions should comply with the specific requirements of different cell types and their usage [18, 24, 25].

In summary, the non-iFBS strengthened certain parameters of the stemness (markers and their maintenance) of bone marrow-derived MSCs such as the enhancement of the formation of colonies (more cells were able to form colonies, and the rate of proliferation was higher in the non-iFBS supplemented medium) in the expansion medium and, simultaneously, the enhancement of the osteo-differentiation potential of the MSCs in the osteogenic medium. Despite the determination of the specific effect of the heat inactivation of FBS on various cells, more detailed studies on this topic will be required. Our results demonstrated both the important impact of the heat inactivation of FBS on the behaviour of MSCs and the fact that it can be highly misleading to compare the results derived from experiments performed under differing culturing conditions in cases where the heat inactivation of the serum is not considered.

### 4.2. The Impact of Various Types of Serum on the Metabolic Activity of the Cells

As previously shown [18, 19, 25], marked differences in cell behaviour have been observed between human serum and FBS. Thus, we subsequently focused on the impact of various types of serum—allogenic human serum (allo-HS), autologous human serum (auto-HS), and serum derived from the donor with the AB blood group (AB-HS)—on the metabolic activity of the MSCs (Figure 3(a)).

Our results indicated that the effect of various types of serum on MSCs is time-dependent, and the reaction of the cells is clearly evident at an early cultivation time point (4 h). The markedly positive effect of the AB-HS on MSC cultivation was not surprising since patients with the AB blood type are considered to be universal serum donors, and moreover, this effect has also been described by other studies concerned with bone marrow-derived MSCs [10] and adipose tissue-derived MSCs [26]. The comparable effects of FBS and HS on the proliferation and migration of human cervical cancer cell lines have been observed, and cell invasion and spheroid formation have been found to be significantly enhanced in the presence of HS [27]. Thus, the effects of HS appear to be cell type-specific.

Conversely, we observed the significant inhibitory effect of all the human sera on the metabolism of the MSCs in contrast to the non-iFBS at early time points (4 and 24 h). This somewhat surprising phenomenon was attributed to the differing quality of the sera (the content of growth factors and immune elements) obtained from the adult donors (the exposure of the immune system to various stimuli during the life span) and the serum extracted from fetal animals (a more naive immune system), as has previously been proposed in other studies [4, 15]. The results presented in Figure 3(b), that illustrate the reduction in the metabolic activity of MSCs cultivated in an adult bovine serum-supplemented medium compared to a fetal bovine serum (Figure 3(b)), support this hypothesis. A further explanation of the inhibitory effect of allo-HS relates to the suboptimal composition of the sera originating from a mixture of blood extracted from different donors (different blood types, ages, and immunological conditions).

In summary, AB-HS appears to be an effective and supportive supplement for the metabolism of MSCs that resembles or exceeds the usage of xenogeneic non-iFBS. Thus, AB-HS should be considered as a promising, in principle safer and ethically more acceptable alternative to non-iFBS with respect to *in vitro* experiments. The research results emphasised the constant conflict and differences between the *in vitro* and *in vivo* behaviour of cells; the *in vitro* results (without any contact with the immune system) indicate that AB-HS is a more efficient provider of metabolic activity to cells than is auto-HS which, in turn, appears to be a significantly less efficient supplement than non-iFBS. Conversely, however, generally under *in vivo* conditions, auto-HS represents the most compatible of the supplements, thus implying that our results should refer only to *in vitro* cell cultivation.

### 4.3. Impact of a Serum Alternative (PR-SRGF) on the MSCs

Platelet derivatives are considered to provide valuable alternatives to FBS, particularly with respect to their potential to enhance the effectivity of the *in vitro* expansion of MSCs [3, 28]. Therefore, the effects were compared of iFBS (even though we previously showed that it is not as potent as non-iFBS) as the standard with a special form of platelet derivative, i.e., “platelet releasate-supernatant rich in growth factors” (PR-SRGF) on the behaviour of bone marrow derived-MSCs.

Despite the observation of the slight inhibitory effect of PR-SRGF on the metabolic activity of MSCs at the early time point (4 h), significant (but limited) enhancement was observed later. Moreover, the significant enhancement was apparent of the cell number during the long-term culturing of these cells in the medium with the PR-SRGF (Figure 1(b)). These findings thus strengthen the potential of PR-SRGF as an appropriate alternative to FBS in the future; while both supplements support the manifestation of certain MSC (stemness) markers, the PR-SRGF enhances MSC expansion (a favourable requirement for the culturing of MSCs) to a greater extent than does FBS. The comparable or enhanced effect of PR-SRGF on certain MSC cellular properties (MSCs derived from bone marrow or adipose tissue) in contrast to that of iFBS has also been described in other studies [10, 26]. Moreover, the FACS analysis of the surface markers of MSCs cultivated in the presence of iFBS and PR-SRGF revealed (Supp. 1) that PR-SRGF tends to maintain the similar expression of selected surface markers on MSCs as does iFBS, a fact that has also been detected in other studies [10, 26]. Although all of these studies revealed the various advantages of the presence of PR-SRGF in the MSC culture, they also demonstrated the necessity of emphasising the critical role of a preparation method involving platelet derivatives and the urgency of compiling a precise definition of the general release criteria that are essential in terms of quality and the enhanced comparability of platelet-based alternatives for future research purposes [3, 15, 28–30]. As Murphy demonstrated, even a small difference in the preparation of the platelet-derived product (e.g., the origin of the platelets) may affect the content, properties, and thus the cell behaviour thereof [26]. Nevertheless, the native variability of MSCs relating to differing cell donors and tissue origin combined with the batch-to-batch variability of PR-SRGF should always be taken into account.

Whereas our results revealed the comparable degree of efficiency of MSCs in terms of forming colonies (the same amount of cells required to be able to form colonies) in the presence of iFBS and PR-SRGF, the sizes of the colonies were found to differ significantly (see the images in Figure 1(c)). While the colonies in the PR-SRGF medium were observed to be large and dense, those in the iFBS medium were much smaller and comprised fewer cells, thus supporting the results concerning increased proliferation following longer term cultivation. Similar results have also been described for bone marrow-derived MSCs [10] and adipose tissue-derived MSCs [26].

With concern to the cell morphology, published studies that focused on CFU-F, the size of colonies, and their morphology are more numerous than detailed studies of changes in single cell morphology following cultivation in various media supplements [10, 18, 19, 29, 31]. We noted slight differences in cell morphology by means of light microscopy (flat cells in the iFBS and concave cells in the PR-SFGF) and apparent differences in the mitochondrial networks in the cells cultivated in the PR-SRGF and iFBS, thus suggesting the differing metabolic status of the cells. Forni et al. demonstrated that the mitochondrial network is dynamic and that its pattern differs according to the various stages of MSC differentiation (nondifferentiated, osteo-, chondro-, and adipo-differentiated) [32]. This implies that the morphology of the mitochondrial network is capable of indicating the state of the cell and its potential tendency to differentiate into a certain direction over time. Despite the undoubted requirement for the more detailed study of this topic, our hypothesis suggesting that differing cell status is conditional on an altered mitochondrial pattern is supported by the elevated degree of the metabolic activity (Figure 3(c)), enhanced oxidative phosphorylation above glycolysis (of approximately 16%), and increased total ATP production rate (of approximately 20%) (Figure 1(d)) of the MSCs in the PR-SRGF compared to those in the iFBS. The trend is distinct despite the high standard deviations caused by the use of different donors. The tendency towards elevated ATP formation in the presence of PR-SRGF could explain the increased long-term proliferation in the PR-SRGF (Figure 1(b)). The presence of iFBS and PR-SRGF affects the metabolism of MSCs on oxidative phosphorylation levels rather than on glycolysis levels, which remain similar with respect to both supplements. This finding is in line with previous studies that mentioned the metabolic flexibility of MSCs with a crucial link to glycolysis and the glucose metabolism [33–35]. According to the results of a study by Mylotte et al., the formation of ATP in MSCs is reduced under stressful conditions (such as hypoxia, ischemia, or glucose deprivation), and moreover, glycolysis has been shown to be the crucial source of ATP for MSCs [34]. Thus, the similar glycolysis rates of MSCs in the presence of the two supplements support the idea of the fundamentality of glycolysis and, thus, its higher stability in cells than that of oxidative phosphorylation. Moreover, the ATP formation rate indicates that the PR-SRGF supplement is capable of mildly elevating the oxidative phosphorylation of cells (i.e., it is capable of increasing the oxidative phosphorylation to glycolysis ratio in favour of oxidative phosphorylation) and may, thus, be less stressful and more proliferation-inducing for the cells. It has been demonstrated repeatedly that cell proliferation is dependent (to a certain extent) on oxidative phosphorylation and mitochondrial electron transport. While the need for oxidative phosphorylation at a certain level has also been observed with respect to cancer cells, the role of glycolysis remains indispensable and crucial for such cells [36–38]; due to glycolysis, the gradually elevated trend of the oxidative phosphorylation of MSCs in the long-term presence of PR-SRGF is able to participate in the enhanced proliferation of cells.

Finally, we analysed the production of cytokines by the MSCs cultivated in the iFBS and PR-SRGF-supplemented media and discovered that the overall cytokine levels in the medium with iFBS (with or without cells) were significantly lower than in the PR-SRGF-supplemented medium. Although extremely high levels of RANTES and EGF were detected in the media with PR-SRGF, the cell cultivation in the media served to slightly lower the overall levels. In addition, decreases were observed in the levels of GRO, angiogenin, and VEGF (in both supplements); EGF and RANTES (in the PR-SRGF); and PDGF-BB (in the iFBS) following cell cultivation indicated the consumption or degradation of cytokines by MSCs, which is in agreement with the results of a previous study [10]. Interestingly, IL-6, IL-8, and MCP-1 predominated in both media following cell cultivation, thus suggesting the production thereof by the cells. IL-8, MCP-1, and RANTES are known to trigger an inflammatory response. Conversely, IL-6 (produced at comparable levels by the cells cultured in both supplements) is naturally connected with impaired inflammatory responses to localised tissue damage or with the impaired induction of acute-phase proteins. In addition, it has previously been stated that “IL-6–producing cells” include bone marrow stromal cells, fibroblasts, and T cells [39], which supports our finding of the comparable levels of the formation of IL-6 by the MSCs cultured in both the iFBS- and PR-SRGF-supplemented media. The question is, therefore, whether the levels of particular pro- and anti-inflammatory molecules (defined by in vitro culturing) play a role or not (the level of IL-6 was one order of magnitude higher than the levels of IL-8, MCP-1, and RANTES) in the prediction of the balance of the immune response. If we assume that it does, PR-SRGF appears to present a higher risk than iFBS with respect to the potential to induce an inflammatory phenotype. On the other hand, it has previously been demonstrated that IL-8 is also linked to the mitosis and angiogenesis of cells [40–42] and that IL-8, GRO, and MCP-1 are produced by carcinoma cells in order to enhance the migratory functions of bone marrow-derived MSCs as a way in which cancer cells are able to support the chemotactic capacity of MSCs [43]. Conversely, these factors serve to predict the advantage of PR-SRGF in terms of inducing an MSC phenotype that supports tissue regeneration, though it potentially raises concerns that such MSCs may also promote cancer growth. A further result that serves to support the use of PR-SRGF concerns its high levels of other cytokines that were determined to be important in terms of cell proliferation. For example, angiogenin and VEGF have been presented as elevators of the angiogenic and cell survival capacity of MSCs [44], and EGF has previously been shown to be an effective booster of MSC proliferation [45]. All of these factors serve to both support and link our finding on the high levels of these cytokines with the high degree of proliferation of cells in the presence of PR-SRGF. In summary, it is evident that PR-SRGF provides a wide spectrum of cytokines, some of which are indeed connected to a proinflammatory response; however, others are associated with an anti-inflammatory function, cell proliferation, and cell survival, in contrast to iFBS. Thus, although PR-SRGF appears to carry the risk of a proinflammatory reaction (which can be induced in the human organism by xenogeneic iFBS), the high potential remains of PR-SRGF to support the proliferative and regenerative capacity of MSCs in cultures and, thus, to enhance the preparation and activation of these cells for the purpose of administration to living organisms as required by clinical practice. Nevertheless, the need remains for further detailed analysis aimed at verifying the safe application of PR-SRGF.

### 4.4. Protein Content Analysis of the Sera and Supplements

The research also involved the analysis of the protein content of the FBS, PR-SRGF, HS, and ABS by means of capillary electrophoresis (Figure 5(b)) (the standard technique used in clinical practice) and the comparison of the levels of the content of the various globulins and albumin. The xenogeneic conditions were represented by two batches of FBS (from different providers), the physiological conditions were represented by human serum AB-HS isolated in our laboratory and commercially obtained HS (AB-HS Sigma), and the clinical conditions were represented by two batches of PR-SRGF.

The analysis revealed three significant differences. Firstly, the values in the measured protein fractions of both batches of the PR-SRGF and the AB-HS were very close to the human serum reference standard. Surprisingly, the commercially obtained AB-HS differed in terms of the measured globulins, which served to provide a warning against its usage. Secondly, a significantly reduced amount of the albumin fraction in the FBS was detected compared to the PR-SRGF and the human serum standard. The role of albumin in the cell adhesion process was previously described in a study by Wei et al., which revealed the predominant adsorption of albumin on hydrophobic surfaces that leads to competition between this protein and cells for presence on the surface; thus, the enhanced content of albumin results in decreased cell adhesion [46]. This phenomenon was apparent in our metabolic activity results at 4 h (Figure 3(c)), at which time the difference between the cells in the FBS and PR-SRGF was observed to be significant. In addition to the differing origin (bovine and human) and the differing nature of the two supplements (serum and releasate), the age and sex factors of the donors may also have played a significant role in the resulting serum albumin concentration [47]. From the clinical perspective, a reduced albumin fraction is typically observed in human serum under inflammatory conditions [48], thus indicating that the PR-SRGF supplement is closer to human serum in terms of its various effects. However, this conclusion is not in agreement with the cytokine assay results, which reinforces the necessity for the further detailed specification of PR-SRGF so as to verify its safe application in practice. Thirdly, while the significantly elevated fraction of alpha-2 globulins in both batches of the FBS and the ABS compared to the PR-SRGF, AB-HS and human serum standard can be linked to the xenogeneic origin of the bovine sera, from the clinical perspective the elevation of this fraction can also be linked to the inflammation process and, thus, to the presence of acute phase reactants such as *α*2-macroglobulin and haptoglobin and the ceruloplasmin content thereof [49]. Hence, this suggests the higher potential of PR-SRGF than FBS in terms of cell culturing and subsequent application in clinical practice.

In summary, all our *in vitro* results served to indicate the preferentially positive effects of PR-SRGF on MSCs with its comparable or higher application potential under in vitro conditions than FBS with respect to its ability to modulate the cell metabolism. Nevertheless, the further detailed investigation of its properties will be necessary so as to verify its safe application in clinical practice.

## 5. Conclusion

The aim of the study was to compare FBS in its common and heat-inactivated form with several easily accessible alternatives that are currently considered promising with respect to *in vitro*, *in vivo*, and clinical applications: human serum (allogeneic, AB, and autologous) and the PR-SRGF platelet releasate which we also characterized using capillary electrophoresis. Our results revealed the significant impact of the heat inactivation of FBS on the MSC metabolism and CFU-F efficiency, thus indicating the need for the precise identification of FBS in publications. We also confirmed differences in the sensitivity of human MSCs to various sera and their alternatives. Our data confirmed that AB serum and PR-SRGF provide efficient and easily accessible alternatives to FBS for MSC cultivation purposes (the analysis suggested that they are comparable or more effective than FBS). Furthermore, PR-SRGF was shown to be an excellent supplement in terms of the long-term effective expansion of MSCs, providing high yields of cells while maintaining their character. We also described in detail the various changes in the morphology, metabolism, and cytokine production of cells cultivated in the presence of PR-SRGF compared to FBS. In summary, our results indicated the extent to which the type of serum (as an essential component for cell cultivation) and its modification are capable of affecting cell behaviour prior to and during experimentation, as well as the degree of urgency of the transition from the application of xenogeneic FBS to that of human serum alternatives in the future for routine research practice and biomaterial and regenerative medicine. We also highlighted the importance of not overlooking common cultivation conditions such as the type of serum and the modification thereof.

## Figures and Tables

**Table 1:** Tested serum types or serum alternatives and their abbreviations.

Serum types or serum alternatives
5% or 10% heat inactivated fetal bovine serum (iFBS)
5% or 10% heat non-inactivated fetal bovine serum (non-iFBS)
10% heat inactivated adult bovine serum (iBS)
10% heat non-inactivated adult bovine serum (non-iBS)
5% allogeneic human serum (allo-HS)
5% autologous human serum (auto-HS)
5% human serum derived from donor of the AB blood group (AB-HS)
5% platelet releasate-supernatant rich in growth factors (5% PR-SRGF)
7.5% platelet releasate-supernatant rich in growth factors (7.5% PR-SRGF)

## Data Availability

Data can be available from the corresponding author upon reasonable request.

## Supplementary Materials

Supplementary Materials Supplementary 1: FACS analysis of MSC surface markers—CD73 in the PE-A channel and CD90, CD14, and CD45 in a FITC channel, CTRL—appropriate isotype control.
