# Supplementary material for: The Impact of Various Culture Conditions on Human Mesenchymal Stromal Cells Metabolism
Source: Stem Cells Int. 2021 Mar 1;2021:6659244. doi: 10.1155/2021/6659244 (PMC7939743; doi:10.1155/2021/6659244)
Supplement: Supplementary Materials — Supplementary 1: FACS analysis of MSC surface markers—CD73 in the PE-A channel and CD90, CD14, and CD45 in a FITC channel, CTRL—appropriate isotype control. [file 6659244.f1.docx]

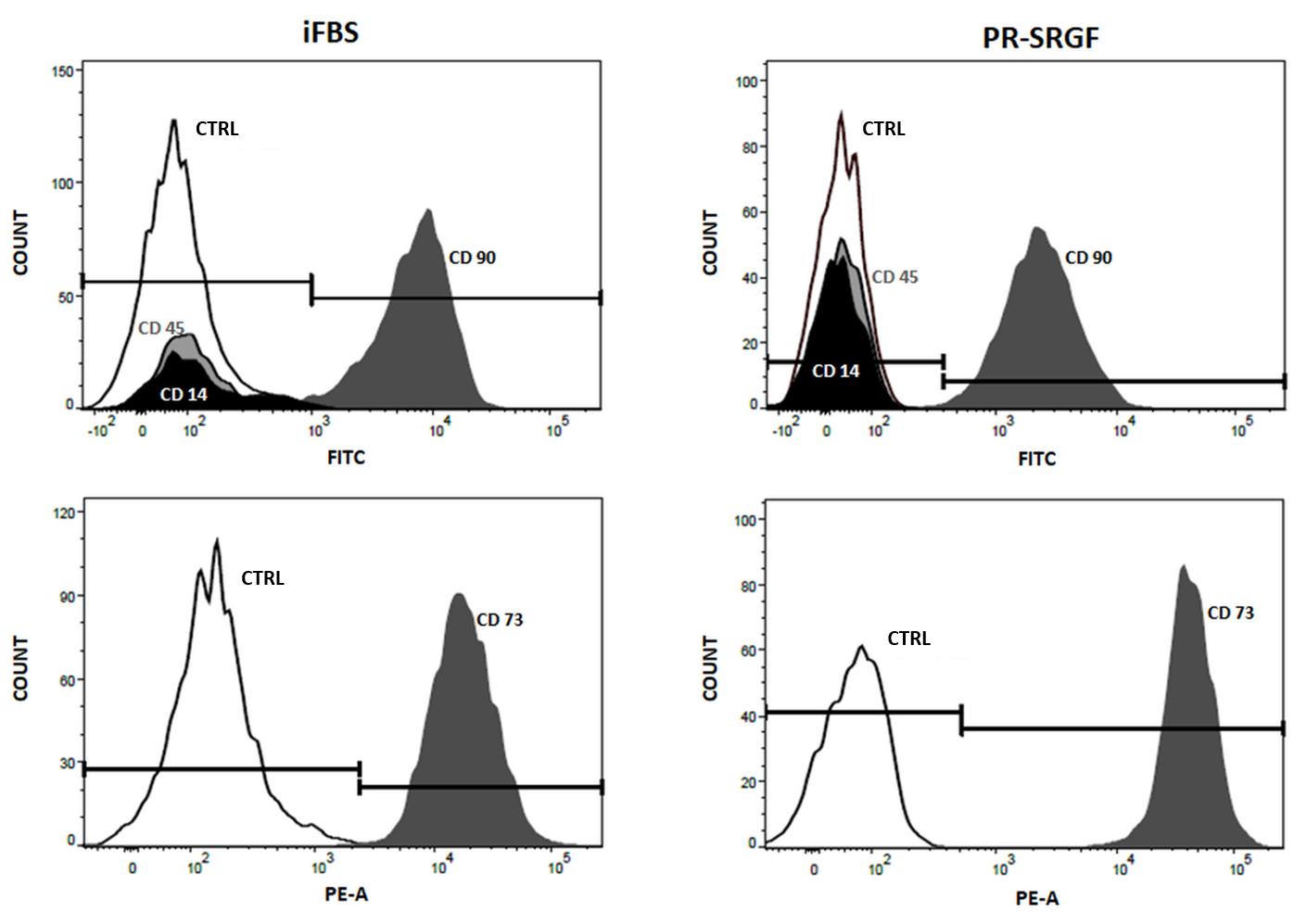


Supplementary 1 – FACS analysis of MSC surface markers - CD73 in the PE-A channel and CD90, CD14, CD45 in a FITC channel, CTRL – appropriate isotype control.
